# Taking Up an Active Role: Emerging Participation in Early Mother–Infant Interaction during Peekaboo Routines

**DOI:** 10.3389/fpsyg.2017.01656

**Published:** 2017-10-10

**Authors:** Iris Nomikou, Giuseppe Leonardi, Alicja Radkowska, Joanna Rączaszek-Leonardi, Katharina J. Rohlfing

**Affiliations:** ^1^Psychology Department, University of Portsmouth, Portsmouth, United Kingdom; ^2^Department of German Studies and Comparative Literature Studies, Paderborn University, Paderborn, Germany; ^3^Faculty of Psychology, University of Finance and Management in Warsaw, Warsaw, Poland; ^4^Faculty of Psychology, University of Warsaw, Warsaw, Poland

**Keywords:** mother-infant interaction, social routines, scaffolding, agentivity, coordination

## Abstract

Dynamical systems approaches to social coordination underscore how participants' local actions give rise to and maintain global interactive patterns and how, in turn, they are also shaped by them. Developmental research can deliver important insights into both processes: (1) the stabilization of ways of interacting, and (2) the gradual shaping of the agentivity of the individuals. In this article we propose that infants' agentivity develops out of participation, i.e., acting a part in an interaction system. To investigate this development this article focuses on the ways in which participation in routinized episodes may shape infant's agentivity in social events. In contrast to existing research addressing more advanced forms of participating in social routines, our goal was to assess infants' early participation as evidence of infants' agentivity. In our study, 19 Polish mother–infant dyads were filmed playing peekaboo when the infants were 4 and 6 months of age. We operationalized infants' participation in the peekaboo in terms of their use of various behaviors across modalities during specific phases of the game: We included smiles, vocalizations, and attempts to cover and uncover themselves or their mothers. We hypothesized that infants and mothers would participate actively in the routine by regulating their behavior so as to adhere to the routine format. Furthermore, we hypothesized that infants who experienced more scaffolding would be able to adopt a more active role in the routine. We operationalized scaffolding as mothers' use of specific peekaboo structures that allowed infants to anticipate when it was their turn to act. Results suggested that infants as young as 4 months of age engaged in peekaboo and took up turns in the game, and that their participation increased at 6 months of age. Crucially, our results suggest that infants' behavior was organized by the global structure of the peekaboo game, because smiles, vocalizations, and attempts to uncover occurred significantly more often during specific phases rather than being evenly distributed across the whole interaction. Furthermore, the way mothers structured the game at 4 months predicted infant participation at both 4 and 6 months of age.

## Introduction

Social interaction requires the coordination of agents' independent behavior in a manner that is appropriate within a given culture, relevant to a situation, and efficient in a task at hand. Whereas, the most important question when thinking about adult interaction seems to be how independent agents come to co-construct a given functional interaction, the focus on the developmental time scale leads us to ask: How do infants become agents in the first place?

Traditional approaches to the development of social skills focus mostly on age-dependent transformations of individual cognitive abilities in children. They view development as a unidirectional trajectory with specific milestones to be achieved on the way toward a particular end point. Viewing agentivity from this perspective positions the process of its development within the infant's mind. These approaches stand in contrast to ecological approaches that focus on continuous individual–environment interactions in which development is bidirectional: Infants not only shape their environment but, at the same time, are also shaped by it. Viewing the development of agency from this perspective means trying to characterize the complex interactional structures in which children are immersed and the transformative role they might possess (Fogel and Thelen, 1987; Reed, 1996). One such approach is the dynamic systems approach, with its notion of reciprocal causality between local and global systems or levels. Reciprocal causality underscores how individual behaviors give rise to and maintain global interactive patterns and how, in turn, they are shaped by them (Riley et al., 2011; Richardson et al., 2014). In the developmental context, one global level seems to play a crucial role in shaping individual skills: the level of structured interaction reenacted for and with the child (Rączaszek-Leonardi et al., 2013; Rohlfing et al., 2016).

Early interactions comprise activities that can be characterized by their high repetitiveness: Repetition of themes (and their modification) occurs not only within single interaction episodes (Stern, 1977; Stern and Gibbon, 1979) but also across multiple interactions in time. In this article, we consider a special form of recurrent interactions, namely social routines. Social routines operate by presenting predictable elements so frequently that the child comes to recognize the structure they constitute. In contrast to coordination through contingent responsiveness to infants' initiatives that are performed locally in a turn-taking manner, social routines facilitate coordination through the predictability of series of caregiver-driven actions as a whole. In well-practiced routines, successive actions follow one another as “moves” distributed between the participants because that particular sequence is given by the format (Snow et al., 1987). The interesting aspect of routines is that it is not crucial for a child to “understand” the individual moves as elements of the routine in order to perform them. For early routines, such as *Hello; How are you? Fine, thanks, and you? Fine*, Gleason and Weintraub (1976) propose that learning the routine does not require knowing what it means to feel fine. This is because the predictability of the appropriate actions provides an adequate basis for the child to perform correctly: It is more about saying and doing the right things at the right time than about any deeper semantic processing. During the first several runs of a routine, the infant's participation might be limited; but, in time, infants learn their moves as well as the roles involved and adults start to demand participation. In this way, responsibility for some parts of the sequence shifts eventually to the infant (Snow et al., 1987; Heller and Rohlfing, 2017). Thus, social routines provide a context in which to observe the development of coordination of activities. Social routines also provide a context in which to observe the process of shaping agentivity, because infants are treated as participants from early on (Ochs, 1988; Zukow-Goldring, 1996; De León, 1998; Takada, 2012; Rączaszek-Leonardi et al., 2013; Nomikou et al., 2016). It is within these interactions that infants learn “to coordinate their engagement, that is, to adjust their behavior in response to and in anticipation of each other's actions” (Rossmanith et al., 2014, p. 3). This happens because the modes of interacting with caregivers instill values of agency (Rączaszek-Leonardi and Nomikou, 2015). Thus, the search for the origins of infants' ability to coordinate with others is none other than the quest for the origins of agentivity within interaction, because interindividual relations shape the individual agents on which they depend (De Jaegher and Froese, 2009).

With respect to the global and local structures shaping agentivity mentioned above, social routines are an ideal context in which to observe how a global format of interactional moves—when repeated often enough—shapes the local behavior of the child; that is, how to perform the correct next step in a sequence and how to act her or his part in an interaction. Given the amount of time caregivers and infants spend every day on various kinds of routines, it might be reasonable to assume that they constitute culturally transmitted practices that scaffold the development of agentivity. Our main goals are, therefore (1) to document the active role infants take so that their actions fit the routine format, (2) to characterize the properties of such routinized interactions that seem to facilitate emergent agentivity of an infant, and (3) to identify whether early in their development infants are engaging in the routine as a whole (orienting toward its global structure) rather than reacting to individual elements of it (acting at a local level).

In this article, we focus on peekaboo play (see also Bruner and Sherwood, 1976) as a restricted “action format” (Ratner and Bruner, 1978; Bruner, 1983) involving a limited number of elements (Ratner and Bruner, 1978) which makes the game easy to repeat. Through repetition, there is a “clear-cut task structure [that] permits a high degree of prediction of the order of events” (Ratner and Bruner, 1978, p. 392). Ratner and Bruner (1978) point to the fact that these games have a clearly demarcated and reversible role structure. Thus, due to its interactive nature, the activity of a peekaboo game not only entails a particular temporal order of individual actions (“what to do next”) and specific junctures (“when is my turn”) but also a particular social organization toward a joint goal: Participants assume certain interactive roles and take responsibility for role-related tasks (“who does what”) (Nomikou et al., 2016). The constituents of the game are the hidden person (mother or infant), the device for hiding (cloth or hands), the agent effecting the hiding, and the agent effecting reappearance. Ratner and Bruner (1978) report that the important *phatic* stages in the game, the presequence and the subsequence, are intended to keep players in contact with each other. According to Bruner and Sherwood (1976), there is a basic “syntax” of necessary constituents: contact—disappearance—appearance—contact. Taken together, the games comprise a global structure in the form of an interaction protocol that can be negotiated between the participants when targeting a joint goal (Rohlfing et al., 2016).

Such early games have been reported to be played when infants are around 2–3 months of age (e.g., Fantasia et al., 2014). They have been characterized as fundamental, allowing the nature of early communication to be explored (Bates, 1979; Bruner, 1983). Fernald and O'Neill (1993) report that during peekaboo, infants show pleasure when they can predict the next step in the actions. However, existing literature has described infants' participation in peekaboo in terms of their ability to change semantic elements: These are, for example, the appearance or disappearance in the sequence (Bruner and Sherwood, 1976; Ratner and Bruner, 1978; Bruner, 1983). These studies showed that, in time, infants understood the semantics of these elements of the game and could vary, for example, who disappears (caregiver, child, or object), how the disappearance is carried out (behind the palms of hands, a cloth, or a chair), or where the reappearance will take place (e.g., same side or different). Other studies have described infants' participation as the production of consistent, speech-like phonological forms in specific phases of early games (e.g., Ratner and Bruner, 1978; Hsu et al., 2014). This is due to the fact that Bruner's and others' original work on peekaboo explicitly related it to language acquisition. The idea is that within such a constrained rule-like interaction format, infants learn to use conventionalized behaviors; that is, not any kind of vocalization but a particular one, and this resembles what happens in language acquisition. Because of the relation to language development, most studies on peekaboo have focused on infants' development of vocalizations within peekaboo routines, investigating infant behavior in the second half of their first year and their second year of life (e.g., Bruner and Sherwood, 1976; Rome-Flanders and Cronk, 1995; Hsu et al., 2014). While taking these behaviors into account convincingly relates early games to later language development (see also Snow et al., 1987; Rome-Flanders and Cronk, 1995), they represent quite advanced forms of participating in a social routine. Ignoring more basic behaviors in research on early games makes infants from birth to 7 months appear passive (Parrot and Gleitman, 1989; Rochat et al., 1999). Clearly, there is a need to develop measures allowing us to assess infants' early participation.

Early participation has also been investigated in experimental setups that manipulated the structure of the peekaboo game. For example, Parrot and Gleitman (1989) investigated 2-, 6-, 7-, and 8-month-old infants' smile, laughter, and eyebrow movements, and Rochat et al. (1999) investigated 2-, 4-, and 6-month-old infants' use of gaze and smile. In both studies, the infants used these modalities when their expectations about the game were violated and/or confirmed. Yet, due to the scripted non-responsive nature of their design, it could be argued that these studies put the infant in a spectator stance (Reddy and Uithol, 2016) in which their participation, although perhaps to some extent observable, was not really demanded. A step away from these controlled observations was taken by Fantasia et al. (2014) who used a semi-experimental setting. The authors investigated infants' participation and expectations in familiar early play routines and in violated forms thereof (no sound or no gesture). Infants as young as 3 months showed overall decreased participation (less smiling, laughing, and body movement) and more stunned face expressions in altered play in comparison to the known play routine. The above studies are interesting, because they show that although infants may not use verbal modalities earlier in development, they are already capable of selecting behaviors from a repertoire of other resources such as smile, body movement, or gaze. Another study addressing the shortcomings of experimental manipulation was carried out by Szufnarowska and Rohlfing (2014). They filmed mothers playing peekaboo with their very young infants in a more natural setting. They found that 2-month-old infants engaged in the activity by smiling back at their mother after she reappeared. The interesting finding from this analysis was that it took more than one repetition of a peekaboo round for the infants to show this response. This underscores the importance of the repeatability of the interaction patterns. Furthermore, for the mother, the smile had an important motivational effect, supporting her in continuing the game. Interestingly, the analyses revealed that infant smiles were, to a large degree, embedded in episodes of mutual gaze. The value of sustaining attention for social interaction with older children has recently been recognized by Yu and Smith (2016). It seems, however, that an interaction with young infants can already benefit from this: In dyads that managed to establish mutual gaze, a smile initiated a series of turns (Szufnarowska and Rohlfing, 2014). These insights clearly speak in favor of the interactive nature of early games in which both participants need to engage. However, in the current literature, the circumstances under which infants gain a grasp of the structure of the peekaboo game are still nebulous. As already mentioned above, existing studies focus on advanced forms of participation, use experimental designs that do not really capture infants' participation in naturalistic environments, and, finally, those few studies that do investigate more natural early interactions have not yet provided a developmental account of early, initial forms of participation. To sum up, although existing results may increasingly lend support to the idea that the global structure is built up, the question *how* infants become capable of maintaining it remains unanswered.

Pursuing the question how infants acquire the global structure of a game, Bruner (1983; see also Ratner and Bruner, 1978) focused on the role of caregivers adjusting to the child's developing sensory and motor abilities and the way this allows a more vivid engagement in and control of an interaction. The argument behind the focus on the role of caregivers is that caregiver scaffolding behavior operates on different timescales: On a short-term timescale, it provides structure to the ongoing interaction. On a long-term timescale, recurring instances or features of the provided structure lead to the emergence and stabilization of interaction frames that shape current and later development (Nomikou, 2015). This is because development is shaped by cumulative experience (Hsu and Fogel, 2003; Fogel et al., 2006), suggesting that variability in the way in which caregivers act on early interactions will be reflected in the later behavior of the infant. This assumption has its roots in socio-cultural theory and (among others) the work of Vygotsky (1978) who suggested that parent–child interaction characterizes development prospectively and is consistent with studies suggesting that different qualities of interactions will lead to different developmental outcomes (e.g., Keller and Gauda, 1987; Bornstein and Tamis-LeMonda, 1997). Bruner and Sherwood (1976) emphasize the caregivers' role in teaching infants the global structure that will result in their more active participation.

Some evidence on the relationship between routine structures and infant participation comes from the work of Ross and Lollis (1987) who found that 9-month-old infants reveal knowledge of the content of a routine and both their roles and those of their partners by taking their turns at appropriate times and by repeating that role during interruptions of the routine. They suggest that understanding aspects of the structure of games may precede the ability or desire to assume certain roles. This, we argue, might underestimate younger infants' abilities to participate. Yet it does provide an interesting approach for looking into early participation and recognition of the global structure of routines by focusing on the individual steps of the peekaboo game and how the infants fit their behavior into these. In concert with the evidence suggesting that sequential structure affects early participation in interactions (Fantasia et al., 2015), it seems plausible that mothers who create more opportunities for their infants to take up their turn will have infants who participate more actively than other infants.

In sum, there is a need for studies that focus on infants younger than 6 months and their communicative means if we are to understand the basis for their increasingly active participation. In line with research on early interactional participation (Rączaszek-Leonardi et al., 2013; Reddy et al., 2013; Fantasia et al., 2014), we do not agree with the statement that infants are “too young to take an active part in…peekaboo” (Rome-Flanders and Cronk, 1995, p. 343). Instead, we argue that interactional behavior in general (i.e., knowing what to do next, the awareness of the interactive role, and how to distribute the work in order to reach a joint goal; see Rohlfing et al., 2016) is a prerequisite of understanding the global structure of the game and the driving force in social coordination. “Many of the forms that later occur in practical situations make their first appearance in the safe confines of structured games” (Ratner and Bruner, 1978, p. 401). Hence, in the present study, we were interested in the development of infants' early participation in a social game and we focused on 4- and 6-month-old infants. More specifically, we were interested in their emerging participation in the routine, manifested in their attempts to take an active role at specific phases of the game as well as the use of social signals within the structured interaction. Given the simple recurrent structure of the peekaboo format, and the fact that previous research has already documented infants' sensitivity to perturbations in the sequence of the actions in the game (e.g., Rochat et al., 1999; Fantasia et al., 2014; Hsu et al., 2014), we hypothesized that infants would attempt to take up an active role at key points in the activity: The use of their behaviors at specific parts of the activity would evidence their sensitivity to the local structure of the game; and their use of different modalities at different parts of the game would evidence their more global recognition of the routine and the role-related tasks. Also, we hypothesized that this participation would increase longitudinally. Furthermore, we predicted that infants' participation would be moderated by the properties of mothers' scaffolding. With scaffolding, we refer to the mothers' way of structuring the activity. This was assessed mostly by the frequency of using specific game phases, although we also explored the duration of the phases as a further possible variable. More specifically, we hypothesized that the way in which mothers structure the activity (e.g., the game phases they use) would relate to the active role that the infants take up in the game. Finally, assuming the cumulative nature of development, we hypothesized that the scaffolding at an earlier age would predict infants' participation at a later time point.

## Methods

### Participants

The data for the present analysis came from a sample of 20 Polish mother–infant dyads (see Szufnarowska and Rohlfing, 2014). We coded interactions of 19 dyads (11 boys and 8 girls) for this study. The data for one dyad was not available for both time points and could not be analyzed. Infants were 4 months old during the first visit (*M* = 126 days, *SD* = 8.79) and 6 months old during the second one (*M* = 186 days, *SD* = 9.63). Participants were recruited in the maternity ward of a hospital in Warsaw.

### Procedure

**Figure 1:**
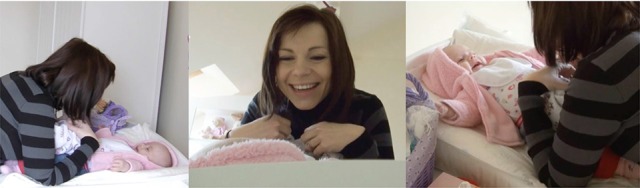
Camera setup.

Data were collected in the families' homes. Mothers and infants were filmed at a temporal resolution of 25 fps with three HD cameras positioned on mountings (see Figure 1). Mothers were asked to place their infant on a table in a supine position and stand in front of her or him. A supine baby position has been shown to enhance mutual gaze (Fogel et al., 1993). The first camera was placed opposite where the mother was standing, filming from below and arranged to capture the mother's face and upper body. The second one was positioned behind the mother, more to one side and registering the infant's face and body from a higher position over her back. The third camera was located laterally on one of the sides, capturing the participants in profile and giving an image of the whole scene (see Figure 1). Sound was recorded through built-in microphones.

The cameras were set up at the beginning of the session. Mothers were asked to play with their infants as they normally do for 3 min, and subsequently to play peekaboo for as long as they wished. The aim of the free play was to familiarize the dyad with the new situation and especially with the cameras. After 3 min, the experimenter reentered the room, asked the mothers to play peekaboo, and left the room once again so as not to distract the dyad. Peekaboo is a social game known and played by Polish mothers (the main phrase “Peekaboo!” translates as “A-ku-ku!”). The mothers were told to play peekaboo any way they wanted to (see Szufnarowska and Rohlfing, 2014). This is a difference between the current study (Szufnarowska and Rohlfing, 2014) and previous studies in which parents were asked to play a rather strict form of peekaboo games (Rochat et al., 1999; Bigelow and Rochat, 2006). When the dyads finished, the mother called the experimenter back into the room.

### Data analysis and coding

We initially familiarized ourselves with the data through repeatedly viewing the videos and collecting single cases that we described qualitatively. This led to the development of coding categories that we then applied to the entire data corpus. To address our questions, we needed to focus on the structure of the peekaboo game and the ways in which (or the resources with which) the infants participated in the peekaboo game.

#### Peekaboo structure

As already mentioned in the introduction, the constituents of the game are the person hidden (mother or infant), the device for hiding (cloth or hands), the agent effecting the hiding, and the agent effecting reappearance. Ratner and Bruner (1978) and Bruner and Sherwood (1976) provide details on the structure of the peekaboo game that we used as an initial guide when viewing the data.

**Figure 2:**
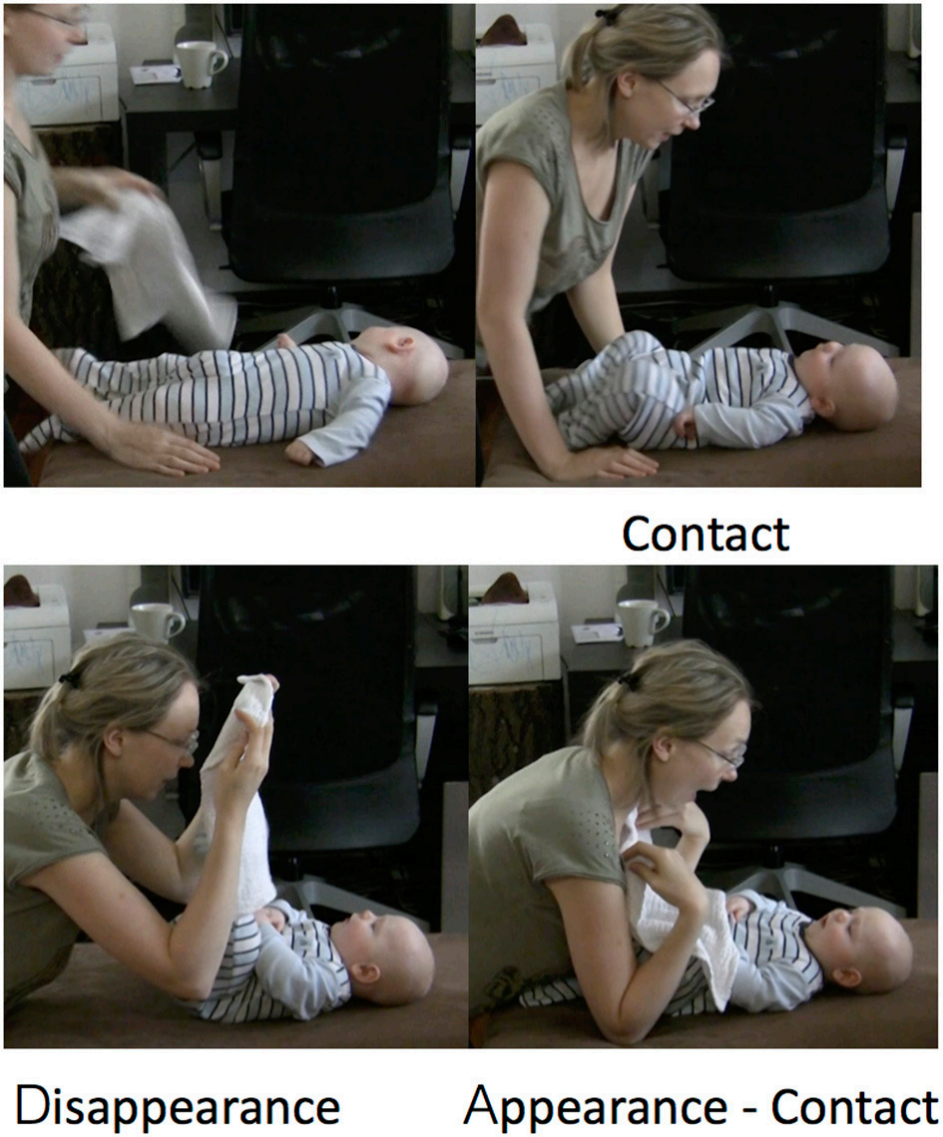
Basic “syntax” of the peekaboo game.

Figure 2 presents the opening sequence of an interaction with a 4-month-old infant. At the beginning of the sequence, the infant is looking toward the side. The mother looms over the infant, touches him, and the infant turns his gaze toward the mother. It is only then that the mother lifts the cloth to cover herself. After uncovering her face, mother and infant resume contact with each other through mutual gaze.

We observed that some caregivers did not allow for variation (initial contact and reestablishment of contact), whereas others allowed for variation of this structure in, for example, the way they carried out the covering and uncovering of the infant.

**Figure 3:**
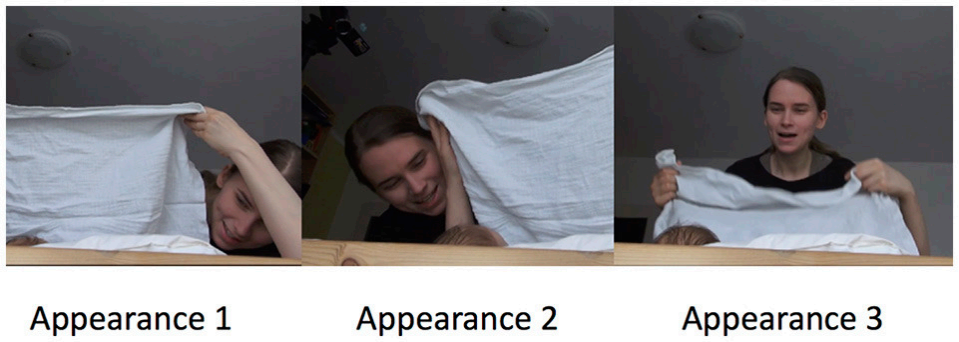
Varying the semantic elements in the syntax.

Figure 3 illustrates three consecutive appearances of the mother. Each time the mother varies the location from which her face reappears. Furthermore, variability could be introduced into the game by varying the duration of uncover from very fast and unexpected to very slow and extended as in the two following examples.

**Figure 4:**
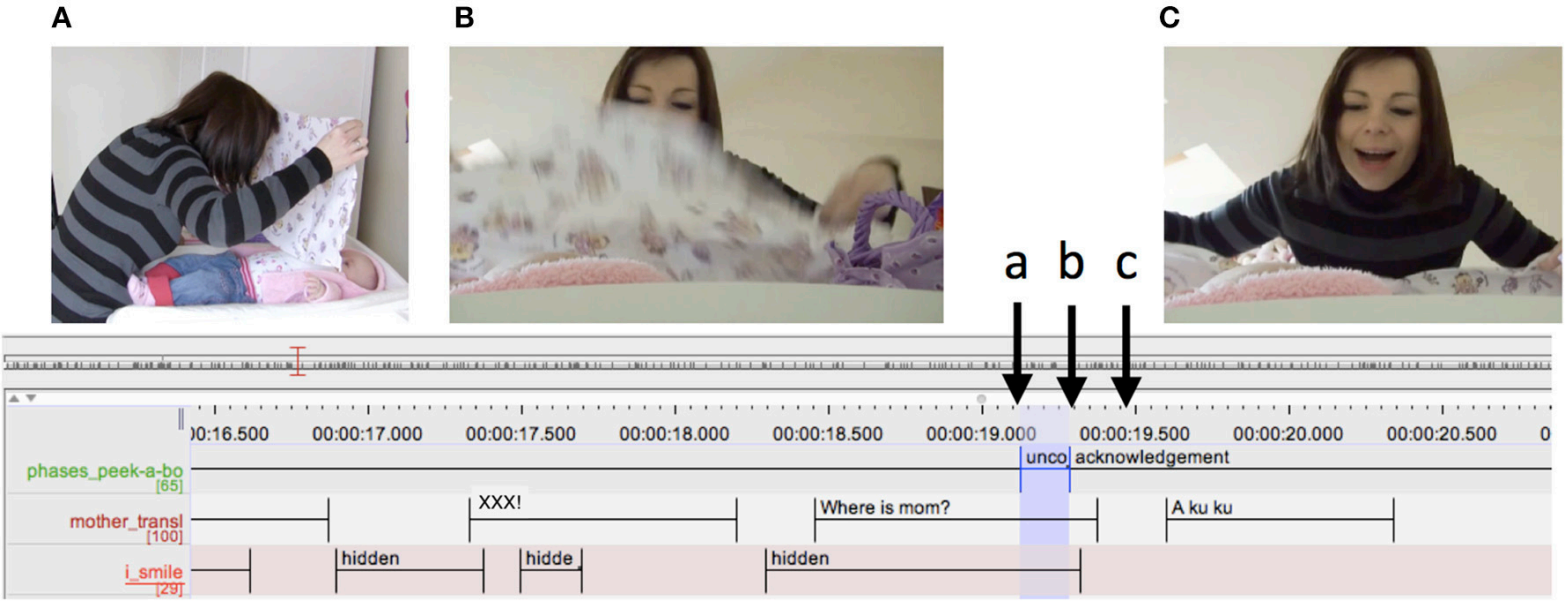
Detail from ELAN transcript. The top tier represents the peekaboo structure. Highlighted in blue is an uncover interval. The letters **(A–C)** refer to the snapshots of the video presented. Arrows indicate the exact moment in time when the snapshots were taken.

**Figure 5:**
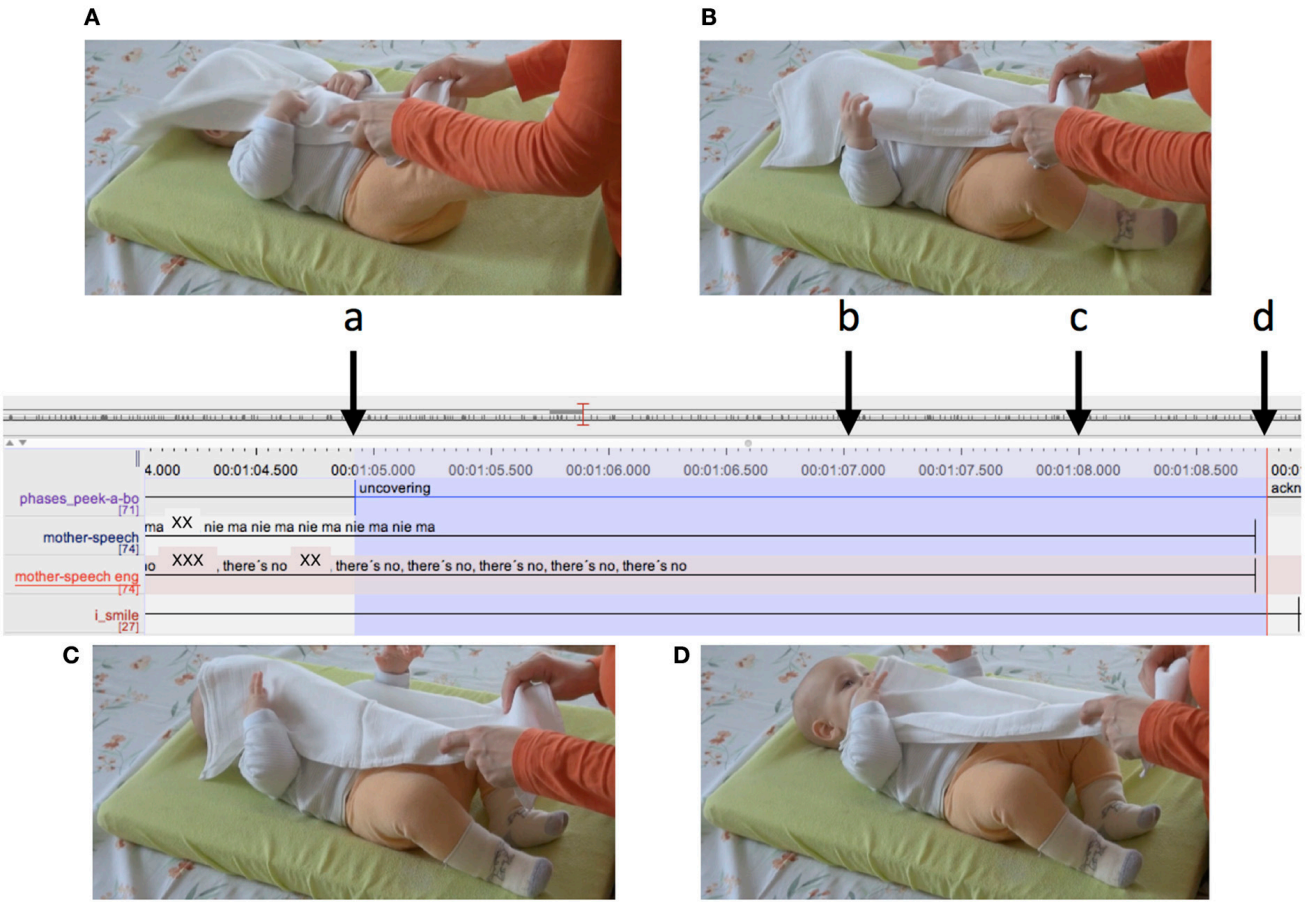
Detail from ELAN transcript. The top tier represents the peekaboo structure. Highlighted in blue is an uncover interval. The letters **(A–D)** refer to the snapshots of the video presented. Arrows indicate the exact moment in time when the snapshots were taken.

In Figure 4, the duration of the uncover phase is around 0.3 s. The mother drops the cloth, looming over the infant to reveal her face. A different case is illustrated in Figure 5. In this case, the mother has covered the infant with the cloth and is stretching the uncovering action, slowly pulling the cloth off the infant's face. Here, the uncover phase lasts more than 3 s.

Further analysis revealed variability in the way the dyads structured the peekaboo. There were cases in which the main constituents were connected with each other through pauses (e.g., at the transition from hiding to reappearance); whereas there were other cases in which this was omitted. We named these intervals “waiting,” in the sense that the mothers were waiting at transition points for the infants to take action, creating slots for infants to take their turn. Yet, mothers actively used these sequences in various ways, so as to engage the infant while their face was invisible to her or him as in the following examples.

**Figure 6:**
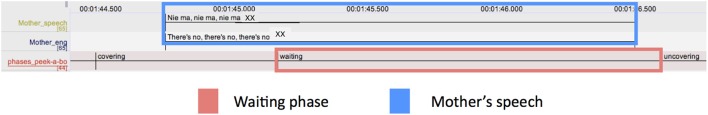
Detail from ELAN transcript. The blue box marks the mothers' speech and the red box the waiting phase of the peekaboo structure.

In Figure 6, the mother accompanies the entire waiting phase with her verbal behavior, pretending she is looking for the infant because she is hidden by the cloth (see transcript below).

P02; 4 months old (01:44–01:46)

1 M: Nie ma nie ma nie ma Asi

There's No There's No There's No Asia

**Figure 7:**
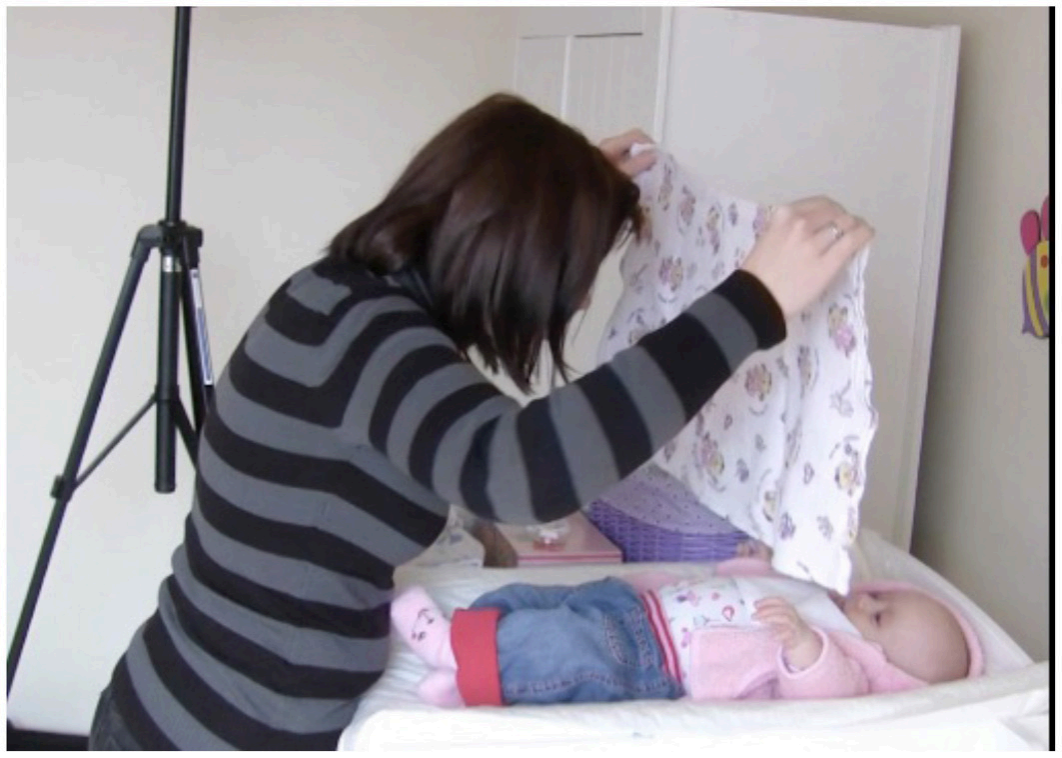
Mother's action on a cloth aimed at sustaining her infant's attention during waiting phase.

In another case, the mother is holding up the cloth like a barrier/curtain between herself and the infant and she moves the cloth from left to right for the entire duration of the waiting phase, sustaining the infant's attention to the location of the mother's face while this is being hidden by the cloth (see Figure 7).

**Figure 8:**
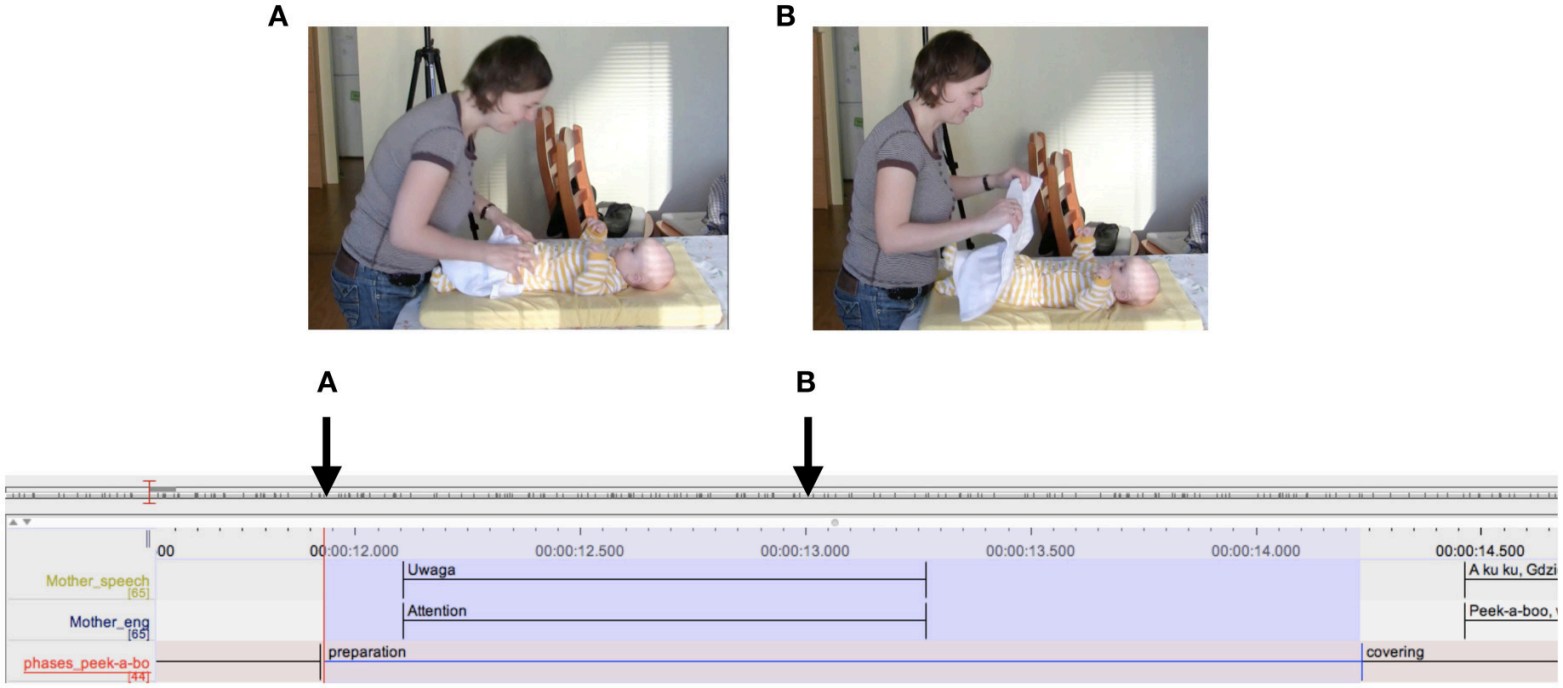
Detail from ELAN transcript. Highlighted in blue is a preparation interval. The letters **(A,B)** refer to the snapshots of the video presented. The arrows indicate the exact moment in time when the snapshots were taken.

A further observation was that mothers sometimes clearly marked upcoming phases of the peekaboo in both their actions and their verbal behavior. In the example below, the mother has unfolded the cloth and is holding it on the infant's body. As illustrated in Figure 8, the mother lifts the cloth to an intermediate position and stops there. She accompanies the lifting movement by saying “Uwaga,” which can be translated as “attention,” thus setting the stage for the next action of covering her face with the cloth. In other similar cases, the mother rearranged the infant's body or the cloth in her hands while asking for the infant's acknowledgment to continue by saying “Jeszcze raz?” (one more time?), or by explicitly announcing the next action by saying, for example, “Teraz mama zniknie co?” (now mummy will be gone, hm?).

We called these types of sequences “preparation” phases, because they somehow mark the upcoming phases of the game and potentially help infants anticipate them. Finally, some dyads inserted other sequences between phases such as tickling games. These differences in structure gave the impression of some peekaboo games being very fast and tightly structured, whereas others were more playful and loose. To account for these differences in the ways peekaboo games were structured, we extended the structure proposed by Ratner and Bruner (1978) and Bruner and Sherwood (1976) (see Table 1).

**Table 1:** Phases of a peekaboo game as performed by the mother.

**Phase**	**Description**
**BASIC PHASES**	
Covering	In this phase, one person disappears (covers oneself or is covered). It starts with the beginning of a movement aimed at covering and ends when the eyes of the person being hidden are covered so that eye contact is not possible; or in a case of a child being covered with a cloth, when the cloth touches the infant's face.
Uncovering	In this phase, a participant is uncovering her or himself (or is being uncovered). It starts with the first movement aimed at uncovering and ends when the eyes are visible and eye contact with the partner is possible.
Acknowledgment	During this phase, eye contact between participants is reestablished. It begins when the eyes of a hidden person are visible again and finishes when another activity starts (e.g., preparation to cover or next covering).
**OPTIONAL PHASES**	
Preparation	In this phase, signs of preparation for the next stage are noticeable. This phase usually precedes a covering phase when, for example, the parent unrolls a cloth or shakes it in preparation for covering the infant.
Waiting	This is a phase between covering and uncovering; here, a hidden person waits to be uncovered or makes an attempt to uncover oneself. It starts when the covering is finished (so eye contact is impossible). If the mother initiates the uncovering movement, the waiting phase ends with the start of this uncovering movement; if the infant initiates the uncovering movement, it ends with the start of the successful movement to uncover. Thus, unsuccessful attempts do not end the waiting phase.
Topic change	In this phase, a dyad is no longer playing peekaboo. This phase is usually a filler between successive peekaboo rounds. The caregiver might, for example, kiss a child, tickle her, or play another social game.

**Figure 9:**
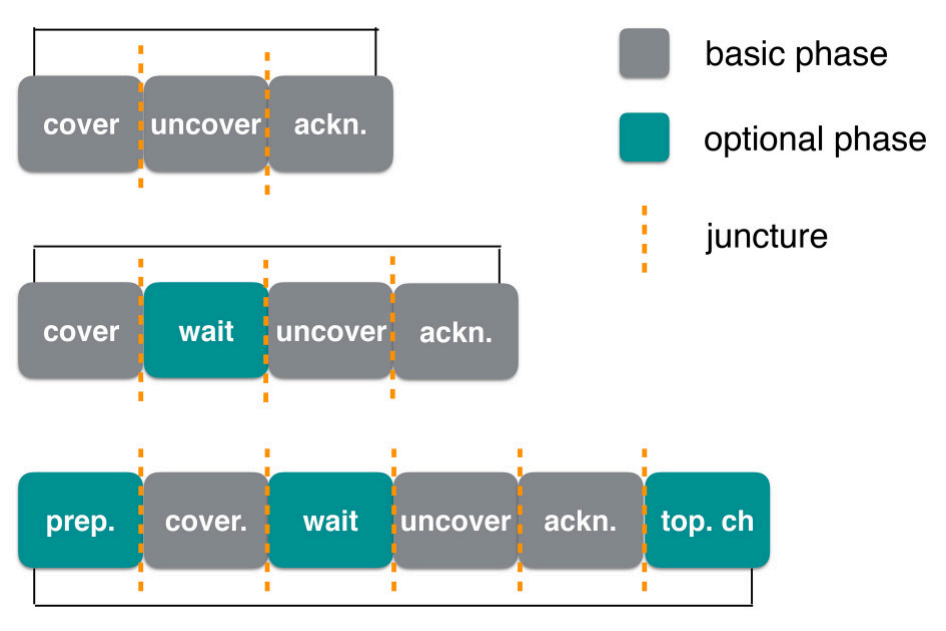
Schematic exemplary representation of the peekaboo phases.

Figure 9 exemplifies different potential structures of peekaboo rounds and how different round structures could provide different opportunities for the infant to participate by fitting her or his behavior to the format of the game. The first round at the top of Figure 9 is a minimal round containing only the basic phases of peekaboo (as in Bruner and Sherwood, 1976). The orange line represents the junctures after which the next phase follows. There are two slots in this minimal round. This means there are two opportunities in which the infant could become active; either after the cover phase, in which the infant would uncover, or after the uncover phase in which the infant could initiate the acknowledgment. The rounds represented in the middle and bottom of the figure are examples of more varied peekaboo rounds. The one in the middle includes a waiting phase after the first juncture. In this case, the addition of this extra phase might prolong the time available for the infant to take an active turn, thus providing more opportunity for her or him. Finally, the round illustrated at the bottom contains an optional round both before the cover and after it. By embedding optional phases before and after the basic constituents of the game, the structure provides more opportunities to participate. It becomes clear that through the inclusion or omission of phases, many variations of the game are possible, both within a specific interaction as well as across multiple interactions and across participants.

Having defined the above types of peekaboo phases, we coded the entire data corpus using frame-to-frame coding of onset and offset of events with ELAN transcription software (Wittenburg et al., 2006). The structure of the peekaboo game was coded in terms of the phases of a single peekaboo sequence—which we call a round of peekaboo—in which one of the participants was covered (mother or infant). The phases of a peekaboo sequence were coded continuously in time and were mutually exclusive. The end of one phase is the beginning of the next. Initially we distinguished between *full* and *short* rounds, with a short round lacking the acknowledgment phase.

The total time of analyzed video material at 4 months was 73 min; at 6 months, 62 min. The average duration of the video recordings at 4 months was 3:49 min (*SD* = 2:30 min). The shortest recorded session was 1:30 min and the longest was 11:57 min. At 6 months, the average duration was 3:14 min (*SD* = 1:17 min). The shortest recorded session was 0:51 min and the longest was 6:40 min.

**Figure 10:**
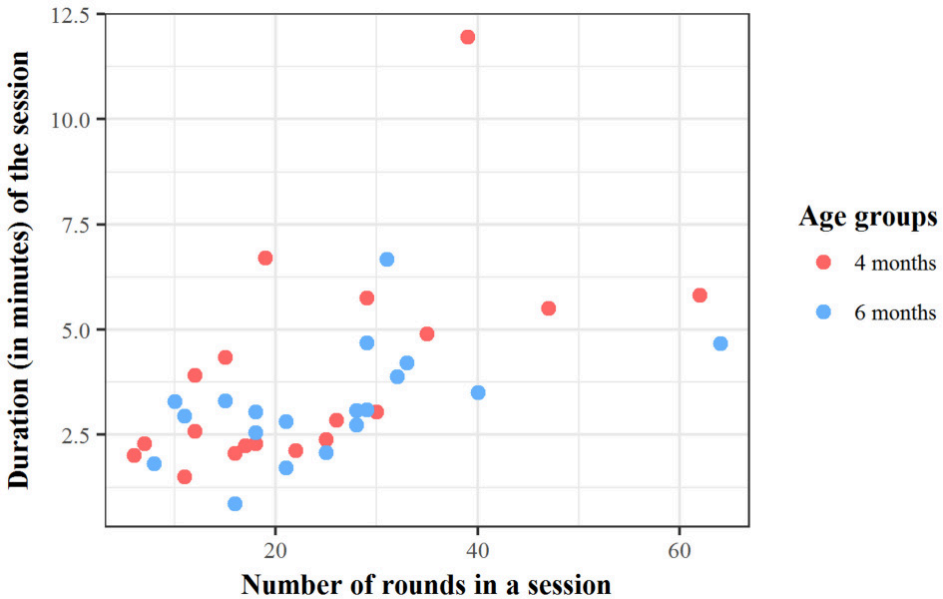
Number of rounds of peekaboo played by every dyad and total duration of video recording sessions.

The total number of rounds played in the Peekaboo game was 925 (448 at 4 months, and 477 at 6 months). The average number of rounds played by the dyads at 4 months was 23.58 (*SD* = 14.36, min = 6, max = 62) and at 6 months it was 25.11 (*SD* = 12.84, min = 8, max = 64). Figure 10 uses a scatterplot to summarize the above data relative to the session duration and number of rounds played by each dyad.

The distribution of the rounds played per dyad (see Figure 10) also illustrates the degree of variability in the way the peekaboo game was structured. Some dyads played the game for a short length of time, whereas others extended the game over longer periods. Also, some dyads played more rounds within a comparable amount of time than others, suggesting that rounds were sometimes performed very quickly and other times at a slower tempo.

#### Infant behavior

**Figure 11:**
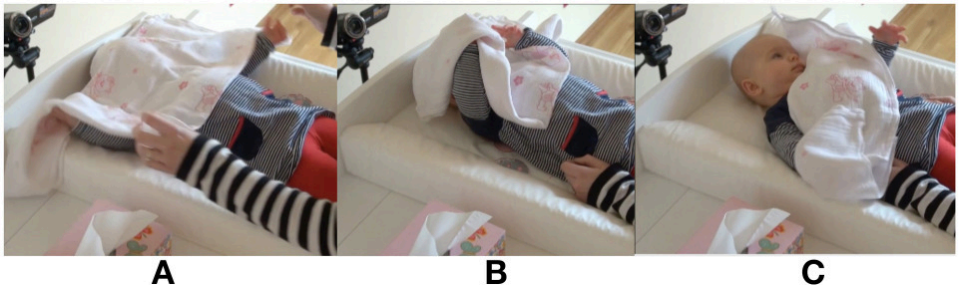
Successful uncovering by the infant at 6 months. Panels **(A–C)** represent the phases of the infant uncover.

Having described the structure of the game, the next step was to observe the ways in which infants participated in it, showing awareness of the game structure by taking an active role by behaving appropriately in the various phases of the game. More specifically, after the covering phase, the infant is required to uncover (either her/himself or the mother); whereas after the uncovering phase, the infant is required to initiate a new cover. Hence, different phases of the game require different actions from the infant. Such a behavior is illustrated in Figure 11: In Figure 11A, the mother has positioned the cloth on the infant's face and releases it from her hands. Figures 11B,C show how the infant then grasps the cloth and manages to pull it downward partially uncovering her face.

**Figure 12:**
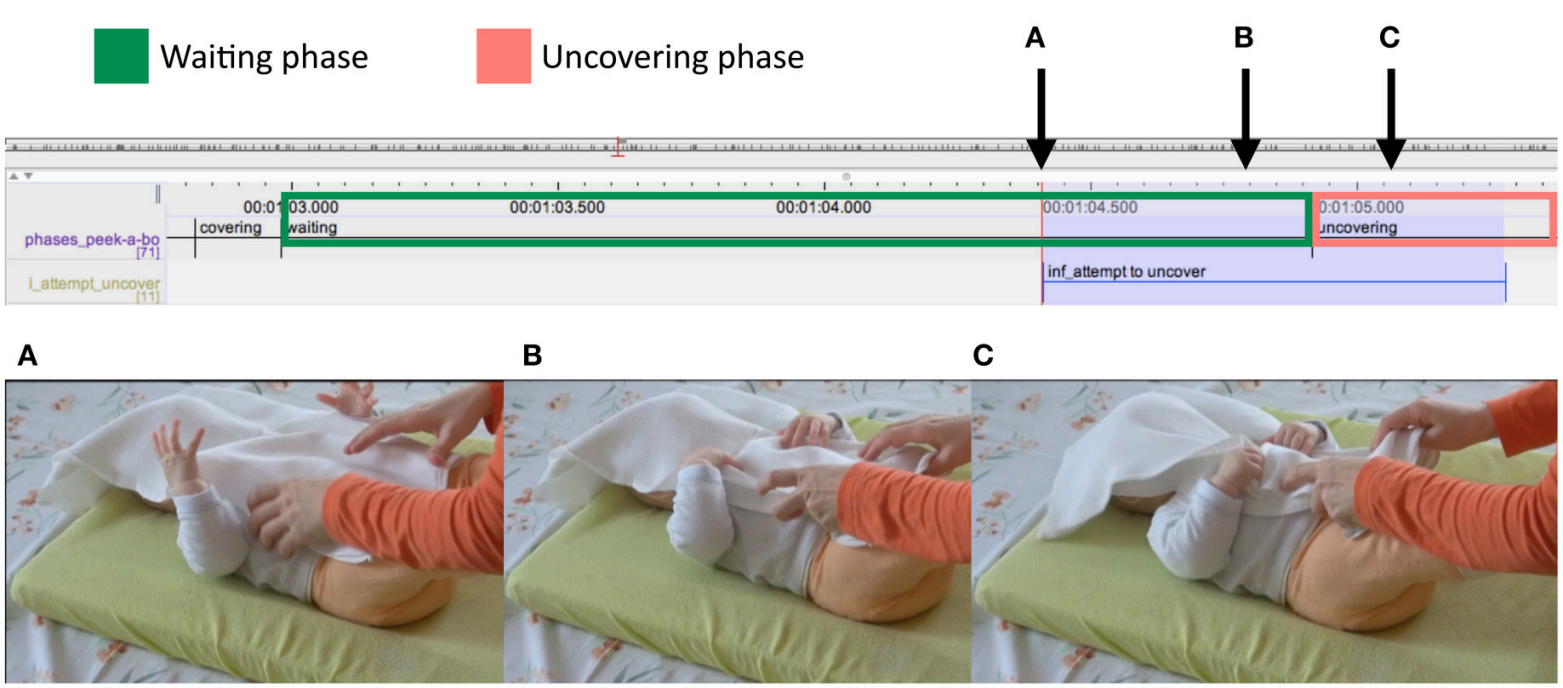
Detail from ELAN transcript. The green box marks the waiting phase and the red box the uncovering phase of the peekaboo structure. Highlighted in blue is the infant's attempt to uncover. Panels **(A–C)** refer to the snapshots of the video presented. The arrows indicate the exact moment in time when the snapshots were taken. Data is from a 6-month-old infant.

A very common observation was that infants often attempted to grasp and pull the cloth, but did not succeed in uncovering themselves on their own. In the case illustrated in Figure 12, we can see the infant attempt beginning in the waiting phase. In Figure 12A the infant is moving her hands toward the cloth, embracing it with open palms while the mother has her hands right on the cloth but is not acting on it in any way. In Figure 12B, which is toward the end of the waiting phase, the infant has grasped the cloth. The mother synchronously grasps the cloth preparing to pull it. In Figure 12C, the mother and infant together pull the cloth, the mother carefully supporting the infant's downward movement.

**Figure 13:**
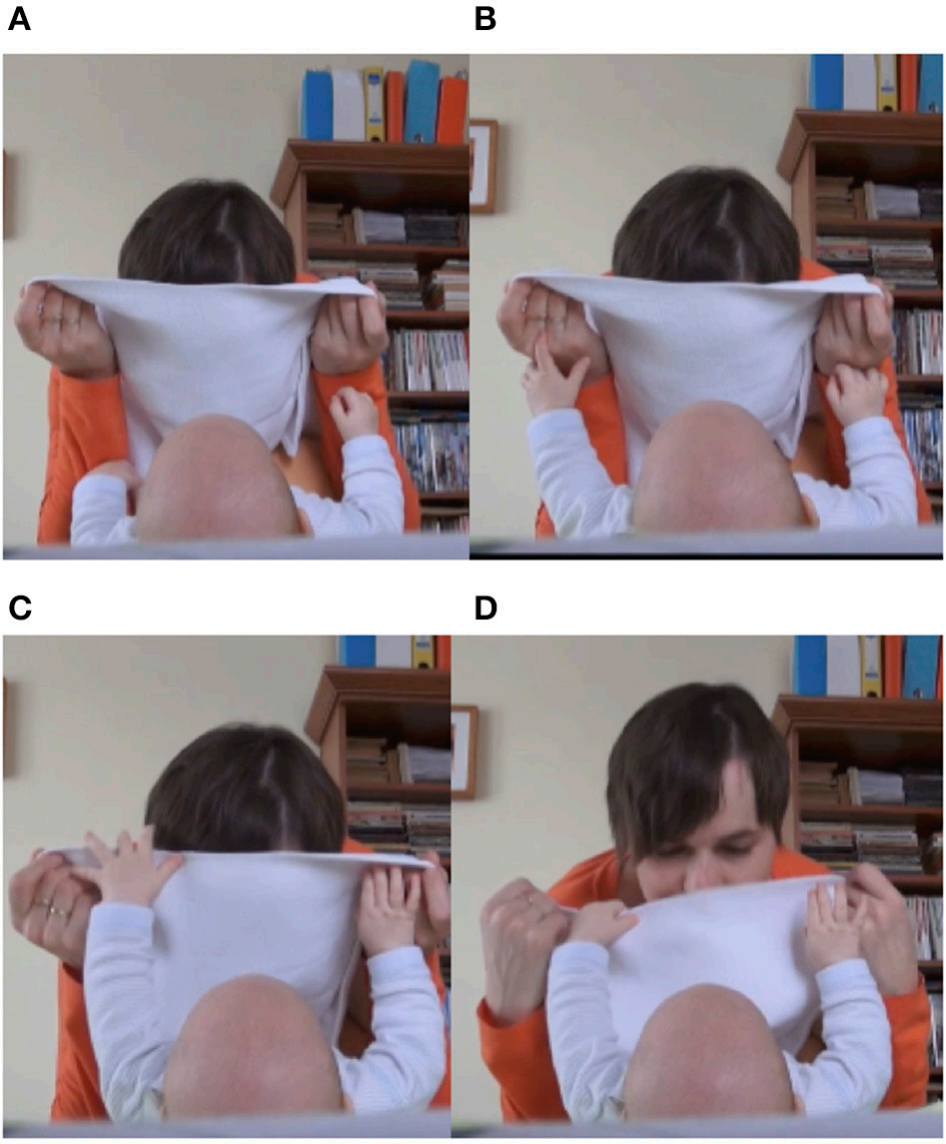
Infant's (6 months old) attempt to uncover the mother's face. The mother is scaffolding the attempt by lowering the cloth as soon as the infant touches it. Panels **(A–D)** represent the phases of the infant uncover.

Another attempt is illustrated in Figure 13. This time, the infant attempts to uncover the mother's face. In Figure 13A, the mother leans forward to enable the infant to uncover her. As a response to the mother's looming motion, the infant stretches her arms and touches her mother's hand (Figure 13B). In Figure 13C, the infant reaches over the cloth to grasp it. At the same time, we can see the mother already starting to lift her head to uncover herself, assisting the uncover. In Figure 13D, the mother, while still holding the cloth, lowers her hand supporting the infant's downward movement and continuing to lift her head upward, she reveals her face.

**Figure 14:**
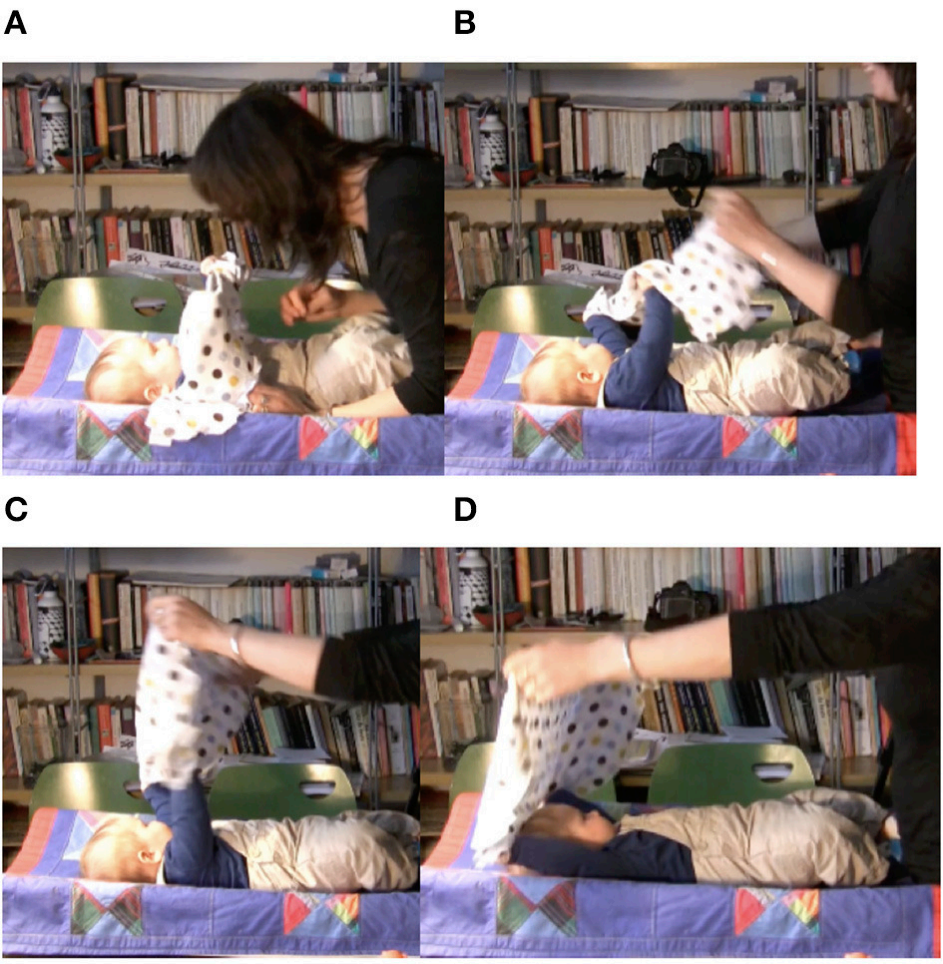
Infant's (6 months old) initiation of cover. The mother is scaffolding the initiative by lifting the cloth to support its transition behind the infant's head. Panels **(A–D)** represent the phases of the infant initiation of cover.

Another infant behavior indicating an active role in the routine is the attempt to initiate or effectuate a new cover. In Figure 14, a 6-month-old infant pulls the cloth over his head. In this sequence, we can once more observe the fine scaffolding of the mother enabling the infant to succeed in the cover. In Figure 14A, the infant extends his arms holding the cloth and he starts moving them backward. The mother facilitates this action by lifting the back side of the cloth (Figure 14B), and following the infant's lead, keeps the cloth raised until the infant rests his arms (and cloth) behind his head (Figure 14D). These observations led to the decision to include infants' attempts to grasp and move the cloth in the right direction in our analysis. An attempt both to uncover and to cover signals participation in the game, even when it is not successful.

**Figure 15:**
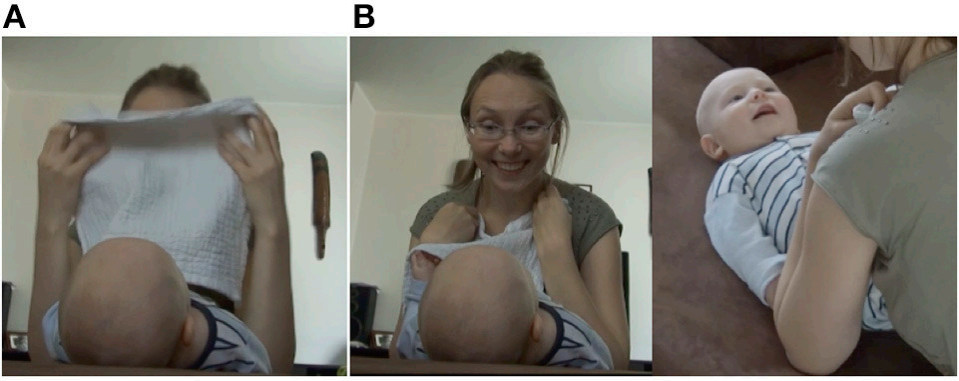
Mother and infant (4 months old) smiling during the acknowledgment phase. Panels **(A,B)** represent the development of the smile.

Participation can also be signaled by other behaviors that the infants can manifest at specific phases. What seems to be particularly relevant is a smile after the uncovering phase (Figure 15) reflecting reestablishment of engagement with the caregiver (Bruner and Sherwood, 1976) or expectation (Szufnarowska and Rohlfing, 2014).

Furthermore, an increased level of vocalizations, appearing in the final phases of the game, also reinforces the reestablishment of engagement after reappearance (Ratner and Bruner, 1978). We then considered such behaviors as additional indices of infants' participation in the game.

The second level of coding, thus, involved the infant's actions in a peekaboo round. It includes infants' responses at key junctures of the activity (Table 2). Here again, the onset and offset of the various coding categories were coded in ELAN. Coding of infant vocalizations was carried out using PRAAT phonetics transcription software (Boersma and Weenik, 2010) and then imported into ELAN.

**Table 2:** Infant's actions.

**Code**	**Subcode**	**Description**
Uncover	Attempt	Behaviors such as reaching toward parent's covered face; touching, grasping, or pulling a cover; moving hands under a cloth while being covered.
	Success	A successful uncovering of the face (own or other's).
Cover	Attempt	Any action that seems to be aimed toward covering oneself or a partner: for instance, reaching hands with a cloth in them in the direction of a parent, throwing a cover in the direction of a parent, pulling a cloth up on one's face, or putting it on one's head.
	Success	A successful covering of the face (own or other's).
Smile		An upward movement of the corners of the mouth; a new smile is coded when there is a pause in smiles of at least 1 s.
Vocalization		Every kind of sound produced by a child including laugh and cry, but excluding vegetative sounds such as sneezing, hiccup, etc.

### Quantitative data analysis

The analytical strategy was (1) to characterize the structure of the peekaboo game, its variability as provided by the mothers, and the variability of infants' behavior quantitatively; (2) to relate infant behaviors to the phases occurring during the game in order to evidence their structuring by the routine; and (3) to use multivariate multiple regression models to check whether the general use and duration of any of the phases of the game was predictive of infant behaviors.

More specifically, in the first step, we calculated descriptive statistics to reveal the structure (the sequencing and duration of phases) of the peekaboo game. Mothers had considerable freedom in structuring and timing the game, and although some phases follow one another logically (e.g., uncovering after covering), they could repeat any of them or introduce some variability both in terms of sequencing and in terms of phase duration. For every round of the game, we registered the duration and sequence of the phases used and computed a frequency distribution of these sequences.

We then turned to infants' behaviors. Every action could occur at different points of the game and more than one time for each phase or round. For every phase in every round, we counted whether a specific behavior occurred at least once, and computed the percentage occurrence of an infant behavior divided by the number of each of the phases. To check whether a specific infant behavior is more likely to occur in a specific phase than in others, which would indicate a recognition of the structure of the game and an infant's active participation, and to check whether this recognition depends on age, we ran a repeated measures ANOVA (one for every behavior analyzed) on this data, using the phase of the game and the age of the infant as within-subjects factors.

We subsequently tested our hypotheses on the relationships among the properties of the reenacted routines and infants' behavior using a multivariate multiple regression model. The number of infant behaviors registered within a session was standardized for each session for every dyad by dividing it by the number of rounds played. The resulting standardized measures constituted the outcome variables of the regression model in which we checked whether they related to the standardized measure of the number of phases used by mothers during the game. The hypothesis was that the relative use of certain phases and their durations might scaffold agentivity. More specifically, the use of the preparation phase might mark the next step in the sequence, allowing infants to anticipate what will happen next. It provides room for an action after the uncover and before the next cover. Moreover, the use of the waiting phase in some way “freezes time.” It stops the game until the infant acts by attempting to uncover her or his face. At the same time, it creates a “slot” for her or his behavior to take place, inviting the infant to act. The acknowledgment phase reestablishes contact between mother and infant and is the phase in which the joint pleasure of playing the game is manifested. In this phase, one would expect the infants to participate by using smiles and vocalizations. Finally, the topic change may provide for an extended period of released tension providing room for the infant to initiate the next peekaboo round. In addition to the relative use of the phases, we checked whether the duration of these phases has a scaffolding effect on infants' participation. Thus, the predictor variables considered in the fitted regression models were in one case, the ratios of the phases; and in another, the average duration of the phases. The outcome variables were the ratios of infants' behaviors.

## Results

### Peekaboo structure

**Figure 16:**
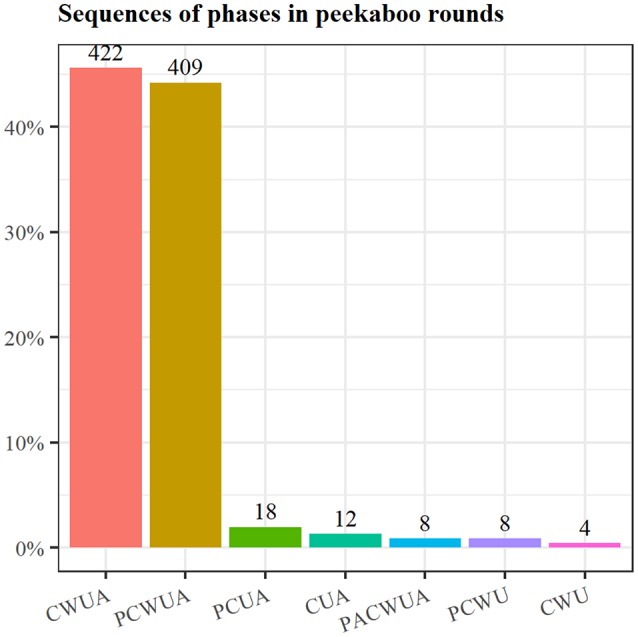
Frequency of the different sequencing of phases realized during all peekaboo games in our sample.

Table 3 presents the count of full rounds and phases standardized over the total number of rounds. Figure 16 uses a bar chart to illustrate the sequencing structure of the rounds played by dyads. Being routinized social games, peekaboo games are certainly quite restricted in the possible sequencing or combination of phases used. This shows up in the frequency of the two most used sequences, which account for almost 90% of the total types of recorded sequences (*N* = 925). In these sequences, together with the three basic phases of the game—Covering (C), Uncovering (U), and Acknowledgment (A)—we always found the Waiting (W) phase in between. The only difference between them was the Preparation phase (P) that was either used or not used at the beginning of the game. This distribution did not differ between the two age groups.

**Table 3:** Ratio of occurrence of phases over peekaboo rounds.

	**Full Round**	**Basic phases within a round**			**Optional phases within a round**		**Other interaction phases**
		**Cover**	**Uncover**	**Acknowledgment**	**Preparation**	**Waiting**	**Topic change**
**4 MONTHS *N* = 19**							
Mean	0.99	1.00	1.00	1.04	0.67	0.97	0.44
*SD*	0.02	0.03	0.03	0.10	0.41	0.05	0.30
Minimum	0.93	0.93	0.93	0.84	0.08	0.83	0.08
Maximum	1.00	1.05	1.08	1.33	1.43	1.00	1.17
**6 MONTHS *N* = 19**							
Mean	0.99	1.00	1.01	1.06	0.64	0.96	0.26
*SD*	0.04	0.03	0.02	0.11	0.39	0.13	0.20
Minimum	0.85	0.91	0.96	0.85	0.06	0.46	0.07
Maximum	1.00	1.07	1.06	1.36	1.46	1.10	0.83

Another observation concerns the distinction between basic and optional phases. The use of the basic phases of peekaboo was quite stable around a ratio of 1 (which means one phase per round of a peekaboo; see Table 3) and with little variation across dyads. At the same time, we found substantial variation in the optional phases: For example, the values for the preparation phase ranged from a minimum of 0.06 to over 1.46 indicating that while some mothers rarely included a preparation phase in a peekaboo round, other mothers used it more than once within a single round. The same holds for the topic change phase. An interesting observation is that the mothers showed little variation in their use of the waiting phase at 4 months (*SD* = 0.05), with both the minimum rate and maximum rate close to 1. Yet, at 6 months, there was more variation (*SD* = 0.13), with some dyads using it <50% of the time (i.e., omitting the use of this phase).

**Figure 17:**
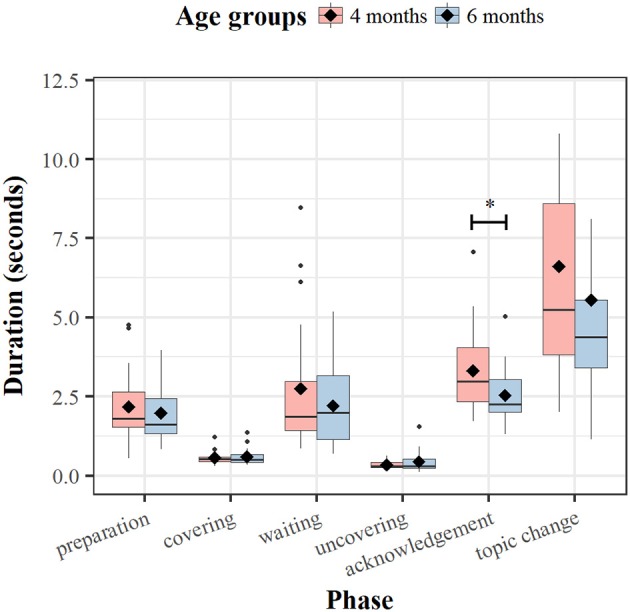
Boxplot of the dyads' averaged duration times (in seconds) for Peekaboo game phases across visits. The black diamonds overlaid on the boxplots indicate the phase mean duration. ^*^p < 0.05. The black dots indicate outstanding observations.

To further explore the way mothers shape the structure of the peekaboo routine, we analyzed the durations of the phases. Figure 17 shows that the covering and uncovering phases were short (lasting around 500 ms) and basically invariant; other phases showed greater variability. The acknowledgment phase, although mothers used it consistently (see Table 3), showed duration variation across dyads. The range in both visits at different ages was quite large with some mothers spending as low as 1.5 s on acknowledgment and others up even to 5 or 7 s As can be seen in Figure 17, a comparison across ages revealed only a minimal difference in the duration of the covering and uncovering phases in the peekaboo games played with 4- and 6-month-old infants. This difference was larger for the other phases. Nevertheless, across the two time points, the average durations of the phase intervals did not differ significantly from each other for any of the phases apart from the duration of the acknowledgment phase [paired-groups *t*_(18)_ = 2.59, *p* = 0.019]. This was shorter at 6 months (*M* = 2.6 s) than at 4 months (*M* = 3.3 s).

### Infant behaviors

**Figure 18:**
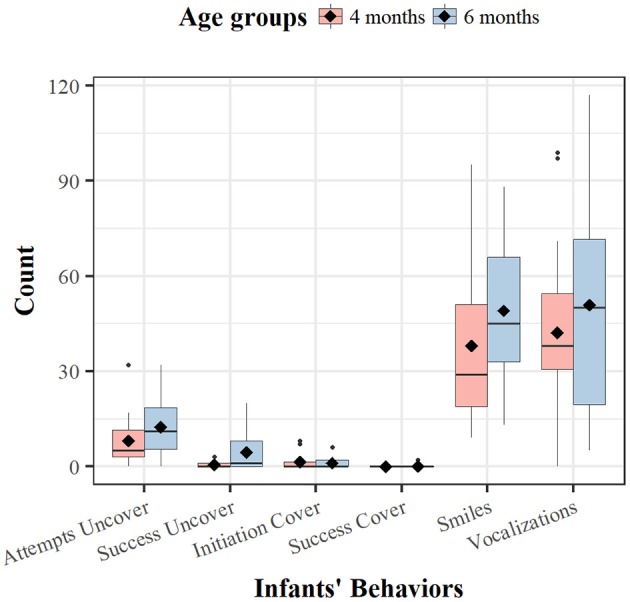
Count of infants' behaviors at different ages. Black diamonds indicate average number. The black dots indicate outstanding observations.

Next, we focused on the extent to which infants react and participate in the peekaboo game by counting the relevant behaviors recorded during the game. Figure 18 presents the average number and variance of infants' coded behaviors within each age group. For behaviors such as smiles and vocalizations, we noticed that already at 4 months of age, they were quite numerous and variably distributed in the various dyads. If we look at behaviors determined more specifically by the context of the peekaboo game, Attempts to uncover were frequent both at 4 and 6 months, whereas the Initiations of covering were indeed quite occasional in both age groups. We observed successful Covering and Uncovering even more rarely; the only exception being the successes in uncovering for infants at 6 months of age.

Given the very low number of Successes in uncovering and covering behaviors (see Figure 18), in all the following analyses, we collapsed Attempts and Successes (to cover and uncover) into the respective categories.

Another observation in these data was a quite systematic increase of activity with age as seen both in absolute values (see Figure 18) and in the frequency of coded infant behaviors standardized on the number of rounds played—that is, the ratios (see Table 4). We used separate *t*-tests on the ratios to evaluate our hypothesis that frequency of behaviors would be greater with age. For Attempts to uncover, *t*_(18)_ = −3.1, *p* < 0.01, and for Smiles, *t*_(18)_ = −2.07, *p* < 0.05, there was a significant age difference, whereas Initiation-of-Cover, *t*_(18)_ = 0.49, *p* = 0.68, and Vocalizations, *t*_(18)_ = 0.39, *p* = 0.64, did not differ in the two age groups.

**Table 4:** Ratio of occurrence of infant behaviors over the peekaboo rounds.

	**Attempts uncover**	**Initiation cover**	**Smiles**	**Vocalizations**
**4 MONTHS *N* = 19**				
Mean	0.40	0.06	1.77	2.27
*SD*	0.27	0.09	0.82	1.58
Minimum	0.00	0.00	0.34	0.00
Maximum	0.84	0.39	3.88	6.17
**6 MONTHS *N* = 19**				
Mean	0.58	0.04	2.10	2.10
*SD*	0.42	0.06	0.69	1.57
Minimum	0.00	0.00	0.62	0.46
Maximum	1.50	0.19	3.00	7.50

Next, we investigated whether infants' behavior related to the particular phases of the peekaboo game. For this purpose, we computed the number of phases enacted by the dyad during the interaction that were accompanied by the various behaviors. For example, if during one interaction, we recorded 10 Preparation phases and the infant smiled in 8 of them, the incidence of Smile during Preparation would be 80%. In this way, we controlled for the variable number of enacted phases in a given interaction. Table 5 shows these percentages averaged across all the dyads at 4 and 6 months. Given that the incidence of every behavior was computed on the total number of each of the phases enacted during the interaction (which varied across phases and dyads), the total of the cells, either in columns or in rows, does not sum up to 100%.

**Table 5:** Incidence of selected infant behaviors in the various phases of the peekaboo game in percentages.

	**4 months**				**6 months**			
	**Attempts uncover**	**Initiation cover**	**Smiles**	**Vocalizations**	**Attempts uncover**	**Initiation cover**	**Smiles**	**Vocalizations**
Preparation	1	0	24	30	0	1	27	31
Covering	4	1	37	9	5	0	46	17
Waiting	30	0	15	38	40	0	23	29
Uncovering	2	0	5	11	7	0	9	8
Acknowledgment	0	3	48	37	0	2	55	41
Topic change	0	3	38	47	0	4	46	44

*If more than one behavior of the same kind occurred within the same phase, only one was counted*.

For each of the selected behaviors in infants (see Table 5), we ran a two-way repeated measures ANOVA with Phase (6 levels) and Age (2 levels) as the two within-subjects factors.

**Figure 19:**
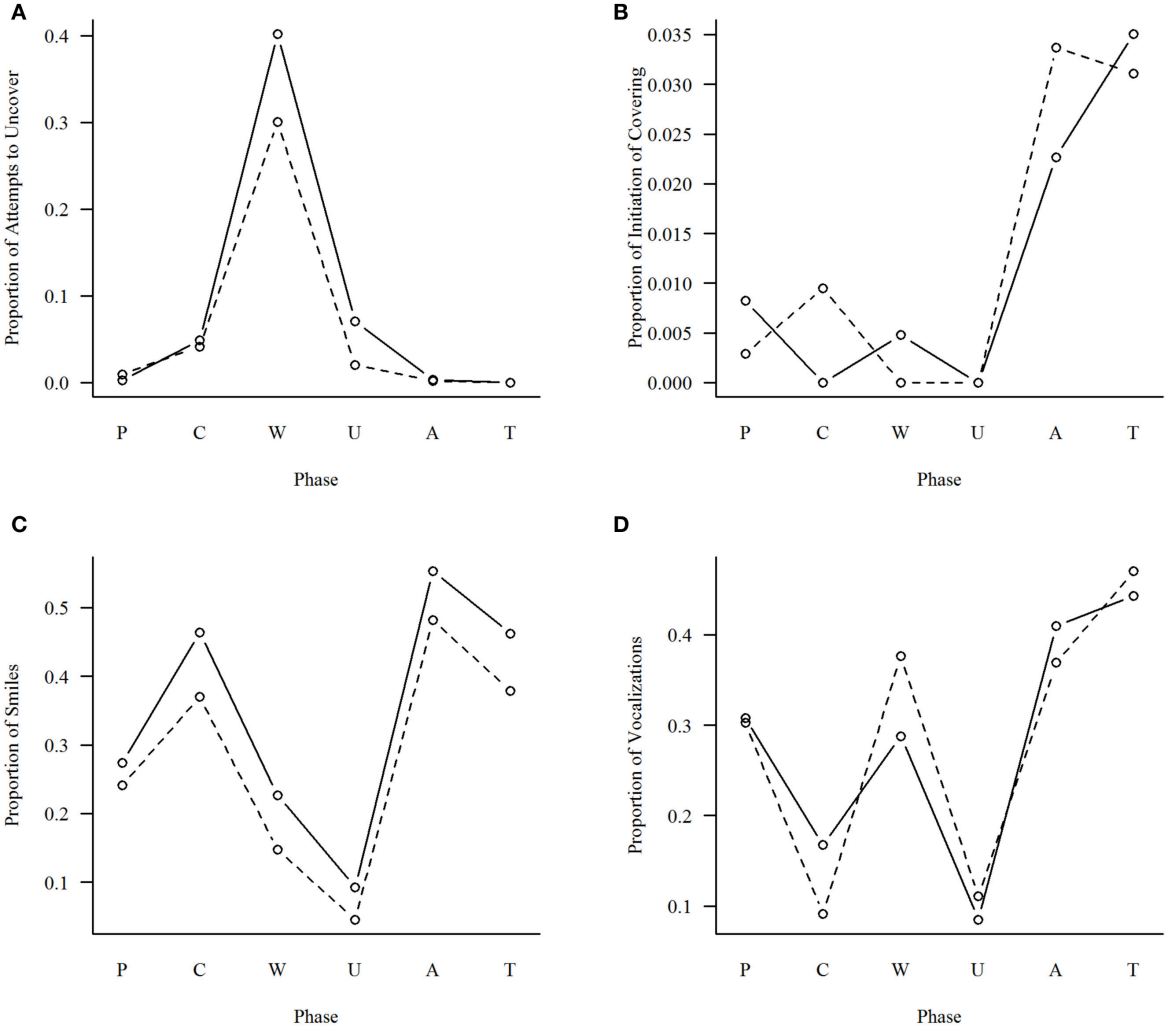
Proportion of phases in which a given behavior occurred. Interaction of Phase and Age. Broken line = 4 months, solid line = 6 months. **(A)** Attempts to uncover; **(B)** Initiation of Cover; **(C)** Smile; **(D)** Vocalization.

For Attempt to uncover, both main effects of Phase, *F*_(5, 90)_ = 40.18, *p* < 0.001, and Age, *F*_(1, 18)_ = 10.95, *p* < 0.01, as well as their interaction, *F*_(5, 90)_ = 2.32, *p* < 0.05, were significant. *Post-hoc* analyses (with Bonferroni correction) clarified the nature of these effects: Attempts to uncover were concentrated clearly during the Waiting phase (significantly greater percentage than in all other phases; see Table 5 and Figure 19), and the significant interaction effect was due to an increased occurrence of this behavior during the Waiting phase at 6 months, whereas in the other phases, there was no change in the incidence of this behavior between age groups.

Regarding Initiation of Covering, the ANOVA indicated only one statistically significant main effect of Phase, *F*_(5, 90)_ = 3.55, *p* < 0.01, but no significant differences in *post-hoc* comparisons. This was probably due to the choice of a very conservative method for the family-wise control of the alpha level (Bonferroni correction).

We then considered the incidence of Smile in the various phases. The ANOVA indicated a significant main effect of Phase, *F*_(5, 90)_ = 20.3, *p* < 0.001, and Age, *F*_(5, 90)_ = 8.56, *p* < 0.01, but no significant interaction. The Bonferroni *post-hoc* analysis clarified that Smiles occurred significantly more often during Acknowledgment, Topic change, and Covering than in the other phases (see Figure 19), whereas they occurred significantly less often in Preparation than in Acknowledgment and significantly more often in Preparation than in Uncovering. Moreover, the same pattern was present at a significantly higher level when the infants were 6 months old.

When looking at Vocalizations, the ANOVA revealed a significant main effect of Phase, *F*_(5, 90)_ = 21.11, *p* < 0.001, but no main effect of Age or any significant interaction. The *post-hoc* analysis indicated that the effect derived from a difference between Covering and Uncovering phases and all the others (see Figure 19), with a significantly lower incidence of Vocalization in these two phases compared to the remaining four.

Taken together, results indicated that infants' behavior was organized by the structure of the peekaboo game, because Smiles, Vocalizations, and Attempts to uncover were found to occur significantly often at specific phases.

### The relation of maternal play and infants' participation

To test the hypothesis that mothers' structuring of the game would scaffold infant participation, we explored the relation between the extent to which mothers used the various phases of the game (their frequency and their duration) and those infants' behaviors that we identified as indices of participation in the game: Attempts to uncover, Initiation of Covering, Smiles, and Vocalizations.

To control for the varying length of dyads' interactions, we divided the number of times a phase was used by the number of rounds played by the dyad. We entered this ratio, computed for all the phases and age (4 and 6 months), into a multivariate multiple regression model as explanatory variables while using the standardized number of infants' behaviors as the dependent (or outcome) variables. Standardization was achieved, as above, by dividing the occurrence of infants' behavior within a certain interaction by the number of rounds played by the dyad—again, to control for the varying number of peekaboo rounds played within interaction.

The test for multivariate model comparisons yielded significant results for the Preparation phase, Pillai's *V* = 0.61, *F*_(4, 27)_ = 10.56, *p* < 0.001, and for the Acknowledgment phase, Pillai's *V* = 0.29, *F*_(4, 27)_ = 2.80, *p* < 0.05, suggesting that these two phases significantly explained the variation in infant behaviors. This means that there was indeed an effect of the predictor variables on the combined outcome of infant behaviors taken together (all four outcome variables at once), and that this was due specifically to the frequency with which the Preparation Phase and the Acknowledgment Phase were used during the interaction. In other words, increasing the frequency of these phases impacted significantly on infants' behavior overall. When we analyzed the outcome variables separately, to determine which of the outcome variables was affected by this effect, the multiple regression models were significant for Attempts to uncover, *F*_(5, 32)_ = 7.34, *p* < 0.001, adjusted *R*^2^ = 0.46; Smiles *F*_(5, 32)_ = 2.77, *p* < 0.05, adjusted *R*^2^ = 0.19; and Vocalizations, *F*_(5, 32)_ = 3.84, *p* < 0.01, adjusted *R*^2^ = 0.28. This finding suggests that some combinations of the phases of the game were related to these behaviors when considered singularly, and the relation was stronger in the case of the Attempts to uncover and Vocalizations, in which the explained variance was greater.

We then turned to check which coefficients in these models differed significantly from zero. In the model with the Attempt to uncover as outcome variable, the only significant coefficient was the one for the Preparation phase, *b* = 0.63, *t*_(32)_ = 4,28, *p* < 0.001. Preparation was also the only significant predictor in the model with Smiles as the outcome, *b* = 0.96, *t*_(32)_ = 2.55, *p* < 0.05, whereas both Preparation, *b* = 2.77, *t*_(32)_ = 3,82, *p* < 0.001, and Acknowledgment, *b* = −5.63, *t*_(32)_ = −2.25, *p* < 0.05, attained significance in the model for Vocalizations behaviors. This means that the presence of Preparation seemed to relate significantly to an increase in Attempts to uncover, Smiles, and infant Vocalizations. Additionally, the use of Acknowledgment seemed to be associated with an overall decrease in Vocalizations.

This model did not yield any effect of age. To explore more closely the relation between the way in which the game was structured at a given time point and infants' participation at a later time, we fitted a new multivariate regression model using the values for Preparation, Waiting, Acknowledgment, and Topic change phases at 4 months of age as potential predictors of infants' behavior at 6 months of age as outcome variables. According to this analysis, both Preparation, Pillai's *V* = 0.72, *F*_(4, 11)_ = 7.19, *p* < 0.01, and Acknowledgment, Pillai's *V* = 0.71, *F*_(4, 11)_ = 6.69, *p* < 0.01, contributed significantly to the multivariate model. However, individual analyses revealed that only one regression model attained significance, namely, the one with Attempts to uncover as the outcome variable, *F*_(4, 14)_ = 5.96, *p* < 0.01, adjusted *R*^2^ = 0.52: Here, the only significant coefficient, was for the Preparation phase, *b* = 0.31, *t*_(14)_ = 3.78, *p* < 0.01. In other words, the use of the Preparation phase at 4 months seemed to be the main variable in maternal behavior predicting the frequency with which Attempts to uncover would be enacted by infants at 6 months.

Another multivariate regression model explored the possible relationship between the duration of the phases used in the game and infant behaviors. The outcome variables in this model were the same as above (e.g., the standardized number of behaviors over the total number of rounds in a session), whereas the predictors were the averaged time durations of all the phases in each dyad and session. In this case, however, the multivariate test for the model comparison did not yield significant results, and this did not justify additional univariate multiple regression analyses on each separate outcome variable. Hence, we did not see any clear relationship between the duration of phases and the frequency of infant behaviors.

## Discussion

The study of human development offers a unique window on the emergence of joint activity formats. By looking at the ways in which infants learn to coordinate their actions in relation to other people we can observe how they come to grasp themselves as agents contributing to a joint goal of an interaction. In many studies, infants' ability to vocalize or make use of conventional means of communication is taken as an indication for their emerging active role in an interaction (Hsu et al., 2014). These include for example the use of vocalizations with specific phonological properties depending on whether the infants are playing games with parents or not. By showing that infants can regulate the types of vocalizations they use depending on interaction context, these studies demonstrate that they have grasped the different interaction structures and how they should behave in them. This, we argue, underestimates infants' early participatory behaviors. By analyzing behaviors which are rather advanced for infants in the first months after birth, existing studies might be making younger infants seem less capable. In our study, we therefore looked for further modalities of early infant participation that could indicate infants' emerging grasp of the structure of social interactions and their role. We examined this by assessing participation in social routines and its development longitudinally. Our goals were (1) to document the active role infants adopt so that their actions fit the routine format, (2) to characterize the properties of such routinized interactions that seem to facilitate emergent agentivity of the infant, and (3) to identify whether early in their development infants are engaging in the routine as a whole (orienting toward its global structure) rather than reacting to individual elements of it (acting at a local level). We also studied the caregivers' ways of shaping this joint activity as predictors of the development of active participation and possible origins of agentivity rather than focusing on the individual mental machinery that makes it possible. As the context for our investigation, we chose a repetitive action format that, as a simple rule-governed activity, enables infants to develop expectancies about the interaction and to display their participation early on. More specifically, we observed mothers and their infants playing peekaboo when the infants were 4 and 6 months old. Guided by existing research on early intersubjectivity and engagement (Markova and Legerstee, 2008; Reddy et al., 2013; Fantasia et al., 2014), we hypothesized that even young infants at the age of 4 months will attempt to take up an active turn in the key phases of the game, and that this will become evident in the modalities they use during particular phases of the game. Extending this research we provided longitudinal comparisons of early routine interactions and expected that this participation would increase with age (Ratner and Bruner, 1978) and that the mother will play a crucial role in scaffolding infants' active participation (Vygotsky, 1978). We therefore explored the relationship between the ways in which mothers structured the peekaboo game and infants' multimodal participation.

The initial exploration of the data provided insights into the multiple ways in which mothers structured the game, varying both the elements of the game, their frequency, as well as their duration. Moreover, infants participated in the game by employing multiple resources: They attempted and succeeded in covering and uncovering themselves or the mother, they smiled and showed excitement through body movement, and they also used vocalizations. From these initial qualitative observations, we developed a coding scheme and operationalized infants' participation as the use of certain behaviors at specific phases of the game: The behaviors included were their attempts to and successes in covering and uncovering themselves or the mother and the use of smile and vocalizations.

Regarding mothers' structuring of the peekaboo, we found little variation across dyads in the use of the obligatory phases of the game (Cover, Uncover, and Acknowledgment). At the same time we found substantial variation in some of the optional phases of the game (Preparation and Topic Change). Comparing the interactions at 4 and 6 months we found that mothers also showed variation in the use of Waiting phase, which at 4 months was used consistently but was more likely to be omitted at 6 months by some mothers. However, the durations of the phases did not change significantly from 4 to 6 months (with the exception of the Acknowledgment phase).

Regarding infant behaviors, we found that already at 4 months of age, all but one infant attempted to uncover during at least one peekaboo round. Successful uncovers were scarce (albeit existing). Attempts to uncover occurred significantly more during the Waiting phase. Interestingly, infants did not vocalize and smile equally across phases, but rather during some particular phases, suggesting a selective contribution according to the structure of the interaction. More specifically, smiles occurred mostly during Covering and Acknowledgment. The use of smiling during Covering could indicate some kind of anticipatory behavior. It could be that the infants recognize what is coming next, be it the tactile sensation of being covered by the cloth or the anticipation of the next phase of the game—namely, the uncovering of the face and engagement with the mother. Infants' use of smile during the Acknowledgment phase indicates their participation in what the Acknowledgment phase is for, namely reestablishing the visual connection with the mother and expressing one's enjoyment of playing the game or simply of being together. At this point we cannot disentangle whether the infant is smiling because of the specific phase of the game or due to the fact that she or he is imitating the mother. Our qualitative observation was that the mothers also smiled during this phase, and that their smiles may actually precede those of the infants. Future analyses should therefore focus on uncovering the fine temporal structuring of mothers' and infants' smiles. It could be that earlier in development the mother uses her smile to elicit a smile, which is a phase-proper behavior. At a later age smiles could appear without a smile from the mother (local cue) but because of the game (global structure). For the vocalizations, we found that they occurred less during Covering and Uncovering. This could be due to the duration of these phases, which were quite short. Yet, the fact that smiles did occur during these short intervals but vocalizations did not, suggests that the intervals were long enough for some reaction but this reaction was less likely to be a vocalization. This poses the question of whether infants chose to use one modality instead of the other. Moreover, the decreased use of vocalizations in these phases is in itself interesting, because it possibly suggests that infants might choose not to vocalize in transitional phases or phases with increased movement. These first results speak in favor of our hypothesis that infants show active participation in the routine by regulating their behavior according to the structure of the game. Furthermore, they point to the fact that the infant may not be locally reacting to the previous behavior of the mother but is sensitive to the fact that different phases of the game require different behaviors. This, we suggest, could be evidence of a more global understanding of the structure of the game as a whole.

Comparing across the two data points, infants' attempts to uncover and smiles increased significantly, lending support to the hypothesis that infants' participation increases as they become familiar with the rules of the game. Concerning vocalizations, we found no difference between the interactions when the infants were 4 and 6 months old.

More crucially, the mothers' way of organizing the game related to infants' behavior. Here, we found that across ages, the use of the Preparation and the Acknowledgment phase significantly predicted the variance in all infants' behaviors. More specifically, the use of the Preparation phase related positively to infants' smiles and attempts to uncover. Also, the use of the Preparation phase together with the phase of Acknowledgment explained a significant portion of variance in infant vocalizing. In this case, the use of the Preparation phase related positively to infants' vocalizations, whereas Acknowledgment made a negative contribution, indicating that the use of this phase was associated with decreased infant vocalizations.

When analyzing the phases at the infants' age of 4 months as potential predictors of infants' behaviors at 6 months, we found that the frequency of occurrence of the Preparation phase was predictive of infants' attempts to uncover. No other regression model involving other phases and infant behaviors attained significance. Finally, analyses exploring the possible relationship between the duration of the phases used in the game and infant behaviors did not yield any significant results.

The results of the multivariate multiple regression models suggest that the structure of peekaboo can be viewed as a kind of scaffold enabling infants to participate. The preparation phases, for example, included mothers' preparing the setting for the cover such as sorting the cloth in their hands or bringing the cloth in position to cover and stopping there. It was this phase that differentiated the two most frequent sequences of peekaboo. Preparation phases can be thought of as initiations of pre-sequences (Schegloff, 1968, 2007). Filipi (2009, p. 3) proposes that their function is to create the conditions for the “entry” of paired actions and to project further action (Schegloff, 1968, 2007). With respect to the structure of peekaboo, Preparation phases project the covering phases that follow. The evidence presented here suggests that the structuring of activities can foster infants' participation in them. Some further evidence on the relevance of structure, has been presented recently by Fantasia et al. (unpublished) who found weaker sequential structuring of early interactions in mothers diagnosed with postpartum depression. Similar to our results with young infants, Hodapp et al. (1984) described mothers' scaffolding behaviors and their effectiveness in early social games for 8- to 14-month-old infants. They reported that in early stages in which the infants had not yet mastered the game, mothers used “attention-getting” and “stage-setting” scaffolds to facilitate play. This behavior has also been observed in other settings such as book reading. Rossmanith et al. (2014) reported on mothers' use of “action arcs,” that is, ways of building up tension at key junctures during the book-reading activity such as just before turning the page. Similarly, Zukow-Goldring (1996, p. 220) in what she called “attention-gathering” interactions, presented an account of how caregivers attract infants' attention to subsequently direct it toward the perceptual structure they have selected. This includes preparatory actions such as an “inbreathe” (Zukow-Goldring, 1997, p. 229; Nomikou, 2015; Heller and Rohlfing, 2017) but also behaviors marking completion of actions, goals, or intermediate action steps (Meyer et al., 2011; Nomikou and Rohlfing, 2011). In our qualitative observations, we did find cases corroborating the findings of these studies. Also, the finding that the Preparation phase at 4 months predicted infants' attempts to uncover at 6 months not only provides additional evidence for the scaffolding role of pre-sequences but also links to the role of long-term timescales of recurring interactions and how these might shape both current and later development (Nomikou, 2015). Yet, it is also conceivable that the use of the preparation phases was regulated by the infants' behavior. For example, it could be the case that the mothers chose to use preparation phases to attract infants' attention to the game before covering when infants were losing interest in the game. We would need to further expand our existing qualitative analyses and quantitative measures to investigate this interactional loop in more detail.

For the phase of Acknowledgment, it is possible that this part of the game concerns the management of child's consolidation processes. It complements the function of the Preparation phase that addresses the child's attention and perception. Ratner and Bruner (1978) considered this phase of reestablishing contact in what they called *phatic* stages of the game to be essential for it to be called a peekaboo game. Our findings suggest that during acknowledgment, mother and infant *share* the experience of playing the game, confirming to each other that they are involved in it; and this confirmation supports infants' agentivity. Alternatively, the acknowledgment phase might also be important for the emotional exchange to establish social attunement (Markova and Legerstee, 2008; Rossmanith et al., 2014). Nonetheless, our findings suggest a negative relationship between the Acknowledgment phase and infants' vocalizations. This relationship is somewhat puzzling. Looking at the descriptive data on the use of this phase, it is striking to see that although all mothers used this phase very regularly, some mothers used it in a much more exaggerated manner (i.e., used more than one acknowledgment phase within a single peekaboo round). There are two possible explanations of this finding: One possibility could be that these mothers might be potentially trying to elicit a response from their infants. In line with the idea of an interactive loop, it is possible that mothers might be trying to create an experience of social attunement to elicit a vocalization if their infant is not vocalizing very often. Another possibility could be that the exaggerated use of the Acknowledgment phase might cause increased verbal behavior on behalf of the mother. In concert with recent work on the development of infants' sensitivity to turn-taking (Gratier et al., 2015) and complementary roles in vocalization (Leonardi et al., 2017) this could lead to infants not vocalizing to avoid overlap with the mother.

Overall, our study not only demonstrated early signs of active participation as possible origins of agentivity in game routines but also attempted to pinpoint some characteristics of the way routines are enacted that might facilitate agentivity. Our findings suggest that the use of some of the phases of the game was indeed related to a higher probability of displaying active behavior on behalf of the infants. For example, the Preparation phase, which is optional to the game itself, turned out to be associated strongly with infants' participation in the game. Likewise, the Acknowledgment phase, which may function on an emotional level, may be used in an attempt to facilitate the way in which interactional practices “draw infants from birth into forms of responsible corporeal engagement” (Takada, 2012; p. 76). When interpreting our findings, we further suggested that infants are probably also playing an active role in regulating the structure provided by their mothers. This speaks to the bidirectionality of the forces shaping interaction. Further research needs to analyze what kind of coordination within which phases of the game is necessary to shape successful game participation.

Most importantly, we can use the research presented here to derive the suggestion that active participation, and thus agentivity, in game routines can be scaffolded by caregivers preparing and acknowledging a peekaboo round. This example contributes to the idea that infants' actions (and development) are embedded in and also generated by the context of caregiving. Expanding from the very constrained setting of peekaboo which we observed, our study could further contribute to understanding how this early form of agentivity could relate to later intersubjective forms such as joint engagement in routines which transcend the here-and-now of the dyad. Although we cannot answer this question directly, our data propose a continuity account for the ways in which more mature forms of agentivity could develop out of repetitive interactions in which an infant first emerges as an agent in relation to others.

## Ethics statement

This study was carried out in accordance with the recommendations of the Ethics committee of the University of Muenster, Germany with written informed consent from all subjects. All subjects gave written informed consent in accordance with the Declaration of Helsinki. The protocol was approved by the Ethics committee of the University of Muenster.

## Author contributions

IN prepared the initial draft. KR supervised the data aquisition. IN, AR, KR, and JR developed the coding schema. IN and AR coded and supervised the data coding. IN provided micro-analytical results while GL together with IN conducted the statistical analyses. All authors analyzed, interpreted the data and wrote the manuscript.

### Conflict of interest statement

The authors declare that the research was conducted in the absence of any commercial or financial relationships that could be construed as a potential conflict of interest.
